# Dignity as a Central Issue in Treating Patients with Dementia Associated with COVID-19 Infection

**DOI:** 10.3390/medicina59091588

**Published:** 2023-09-01

**Authors:** Sorin Hostiuc, Eduard Drima

**Affiliations:** 1 Department of Legal Medicine and Bioethics, Faculty of Dental Medicine, Carol Davila University of Medicine and Pharmacy, 050474 Bucharest, Romania; 2 Medical Clinical Department, Dunărea de Jos University, 800201 Galați, Romania; drima_eduardpolea@yahoo.com

**Keywords:** COVID-19, pandemic, bioethics, dignity, dementia

## Abstract

COVID-19 was initially considered a primary respiratory disorder associated with various short- and long-term complications, affecting many patients and imposing a significant burden. Patients who have dementia are especially vulnerable to the SARS-CoV2 infection, which is associated with an increased risk for neuropsychiatric complications. These patients need a unique approach to managing ethical issues related to the COVID-19 pandemic, including autonomy, veracity, non-maleficence, justice, compassion, and dignity. The latter is one of the most elusive and misunderstood concepts in medical ethics and is extremely important in debates surrounding the proper management of patients with dementia. However, it is often left out of ethical analysis, as most clinicians, when debating issues associated with clinical practice, often evaluate only the “classical” principles of biomedical ethics. In this article, we aim to assess the unique features of dignity in treating this group of patients during the COVID-19 pandemic. We will briefly analyze dignity as a bioethical concept. We will further explore its principal axes, namely compassion, creating a humane and purposeful environment, employing persuasion to meet the person’s essential needs, exerting a certain degree of mild restraint to meet the person’s critical needs, minimizing harm in terminal care, and justice, through the lens of people who had dementia during the COVID-19 pandemic. Applying this principle in clinical practice requires significant commitment from all healthcare workers. New approaches to the analysis of dignity, such as through the Ring Theory of Personhood, may facilitate its understanding by practitioners and aid its implementation in populations with multiple vulnerabilities, such as dementia patients, during an infectious outbreak that generates significant social and medical changes.

## 1. Introduction

COVID-19 is associated with various neuropsychiatric complications that affect many patients and impose a significant burden on healthcare systems worldwide [1,2]. A study involving 154,068 COVID-19 patients and over 11 million controls found that there is a significantly increased risk of memory and cognitive disorders, ischemic and hemorrhagic stroke, peripheral nervous system disorders, and episodic disorders such as migraine, Guillain–Barre syndrome, encephalitis, and encephalopathy in the post-acute phase [3]. COVID-19 has been found to accelerate the progression of dementia in patients with pre-existing conditions and to cause new white matter injuries, indicating decreased resilience to new insults [4]. They may be related to increased vascular injuries [5]. In elderly patients, COVID-19 has been shown to cause dementia-like symptoms in approximately one-third of cases, a significantly higher proportion than the general population of the same age [6]. A study of nearly 1.5 million subjects found that the risk of dementia and mortality was significantly increased in older adults after two years of follow-up [7].

Some subgroups of patients with dementia are especially vulnerable to COVID-19 infection and are associated with an increased risk for neuropsychiatric complications. For instance, a study by Wang et al. found that African American dementia patients have an almost threefold increase in the adjusted odds ratio of becoming infected with COVID-19 [8], and their prevalence is nearly double compared to white American patients (19% vs. 10%) [9]. In a scoping review, Udoh et al. showed that African American patients with dementia and COVID-19 had longer delays in accessing healthcare services, including ICU access, mechanical ventilation, or transportation [10]. In Europe, the Roma population has been suggested to have an increased risk of COVID-19 [11], but clinical results are inconclusive. Mocanu et al. found that Roma patients had more risk factors for mortality after orotracheal intubation, but overall mortality did not increase [12]. Migrants, mainly undocumented migrants, healthcare workers, and those living in camps, were found to be significantly more likely to be infected with SARS-CoV2 than other population groups [13].

All these characteristics of patients who have dementia require a unique approach to managing ethical issues related to the COVID-19 pandemic, including matters about autonomy, veracity, non-maleficence, justice, compassion, or dignity. When debating ethical issues associated with clinical practice, most clinicians often evaluate the “classical” principles of medical ethics, namely autonomy, beneficence, non-maleficence, and justice. Dignity is one of medical ethics’ most elusive and misunderstood concepts [14,15,16,17,18]. As its importance is extremely high in debates surrounding the proper management of patients with dementia [18,19], we aimed to evaluate its unique features in treating this group of patients during the COVID-19 pandemic.

## 2. Research Methodology

The evaluation of the way dignity was included in the ethical analysis of dementia patients during the COVID-19 pandemic was based on a systematic study of the results obtained during searches in the Web of Science database. We used the following keywords: “dignity + COVID + ethics”, which yielded 58 results; “dignity + COVID + bioethics”, which yielded 12 results; and “dignity + COVID + dementia”, which yielded seven results. We analyzed the relevant articles as determined by the abstract analysis, and full papers were downloaded if the abstract contained significant, related data. We then evaluated the list of references for each downloaded article to identify other potentially valuable articles. We also extended our analysis of general ethics literature regarding recent developments in the ethical analysis of dignity.

## 3. Overview of Dignity as an Ethical Concept

As a general concept, dignity means the quality of being respected or charged with moral authority [20]. It is one of the least understood but still widely applied bioethical concepts in areas such as elderly care, assisted reproductive technologies, psychiatric care, or human enhancement. Schulman found four primary sources of human dignity [21].

In classical antiquity, dignity was seen as synonymous with excellence and distinction. In the Biblical religions, it was seen as being generated by the unique position of the human being in the natural world, as humans were made in God’s image. In summary, Christianity brings another dimension to human dignity, more anthropological [22], through an increased emphasis on the creative Divine work and the intrinsic goodness of its creation, especially in human beings [23].

Kant’s moral philosophy saw dignity as inherent worth, which applies to all human beings and only to them due to their capacity to reason and act autonomously based on their reasoned will.

The fourth source is represented by 20th-century international declarations, such as the Charter of the United Nations and national constitutions [21]. In the Charter of the United Nations, dignity is introduced since in the first article: “We the peoples of the United Nations determined to have succeeding generations from the scourge of war, which twice in our lifetime has brought untold sorrow to mankind, and to reaffirm faith in fundamental human rights, in the dignity and worth of the human person, in the equal rights of men and women and of nations large and small, and to establish conditions under which justice and respect for the obligations arising from treaties and other sources of international law can be maintained, and to promote social progress and better standards of life in larger freedom (…)” [24]. Similarly, in the Charter of Fundamental Rights of the European Union, the first article refers to human dignity by stating, “Human dignity is inviolable. It must be respected and protected” [25]. In the explanation of the article, human dignity is considered not only a fundamental right in itself but also the factual basis for fundamental human rights, meaning that other human rights must be respected but not to the extent they cause a breach of dignity. The latter must be respected if dignity opposes another human right [26].

As with its sources, the current concept is still highly heterogeneous. Ashcroft summarized the current views in four main categories: some authors see dignity debates as incoherent, unhelpful, and misleading; others find some usefulness in the concept, but it is usually reduced to autonomy; a third sees it as a concept in a family evaluating capabilities, functioning, and social interactions; and the fourth sees it as a metaphysical property of all moral agents, serving as a foundation for human rights and morality [27]. Moreover, various authors have described different types of dignity associated with clinical practice, of which the most important for this study are intrinsic and moral dignity. The person’s ethical behavior generates moral dignity; it arises when the moral agent acts virtuously and is associated with moral excellence [20]. Its source is mainly found in classical antiquity [21].

Intrinsic dignity refers to the value of human life, determined by its quality of being a moral agent, independent from individual characteristics such as age, gender, education, intelligence, etc. [20]. It is always present in every human being, cannot be lost, and has no intermediate degrees. This approach stems from the Kantian concept of dignity, which applies to all human beings and always requires treating people as ends in themselves [28]. This is the most widely used approach to dignity in clinical medicine and bioethics, as it generates specific obligations for healthcare professionals. The quality of care depends crucially on their ability to assign intrinsic value to each patient and to see them as dignified and worthy of respect, irrespective of the actual diseases of the patients, their social status, race, gender, or any other characteristics. Some of them are in need of not only managing their medical needs but also maintaining some of their essential physiological functions, such as hydration, nutrition, or hygiene.

A more recent approach to evaluating dignity was presented by Chua et al. [29] in a scoping review regarding the perception of dignity by patients through the so-called Ring Theory of Personhood [30], which sees it as a series of concentrical circles, each external one fully encompassing the internal one, starting with the innate, followed by the individual, relational, and finally societal rings. The innate ring is embedded in the belief that all human beings deserve to be seen as persons and contains personally identifiable characteristics appertaining to and unique to each person, such as name, gender, family identity, religion, and cultural uniqueness. The individual ring is generated by personal beliefs, preferences, norms, values, and principles based on innate features but can be modified through will. The relational ring includes all the relationships a particular individual constructs, shaping its beliefs, values, and principles through personal interactions. Finally, the societal ring consists of the social, religious, professional, legal, and institutional expectations of us [29].

During the COVID-19 pandemic, human dignity has been thoroughly evaluated concerning death, dying, and end-of-life care [31,32]. Dying with dignity was often deprioritized as healthcare systems emphasized live-saving measures without significant evaluation of unforeseen consequences, leading to discrimination (in healthcare access, age-related, and so on), little regard for the quality of life, subjective allocation of finite healthcare resources, and limitations of liberty or autonomy [33,34,35,36,37]. Another area in which many authors have analyzed dignity during the pandemic was represented by its effects on vulnerable groups, such as migrants [38,39,40], the elderly [33,41], victims of domestic violence [42], people with disabilities [43], or even the treatment of the relatives of the deceased [44].

## 4. Analysis of Dignity in Dementia Patients during the COVID-19 Pandemic

Tranvag et al. found, in a meta-synthesis, the following issues to be central to the ethical analysis of dignity in dementia care: compassion, creating a humane and purposeful environment, employing persuasion to meet the person’s essential needs, and exerting a certain degree of mild restraint to meet the person’s fundamental needs [18]. These are of utmost importance during the pandemic, as are minimizing harm in terminal care [19,45] and justice [33].

Compassion is defined by the presence of a behavioral disposition to act in a way that facilitates and enriches the finality of the medical intervention by understanding the suffering of the patient [46]. In patients with dementia, compassion can be viewed through three main axes: connection, respect, and care [47]. Connection means being able to identify and recognize the suffering of the patient and to communicate an understanding of this suffering [47]. As a general rule, this personal connection is challenging for dementia patients. It is further complicated when associated with COVID-19, as, at least in the early stages of the pandemic, physicians took significant hygienic precautions in dealing with patients. A healthcare worker using masks and face shields has objective difficulty revealing compassion, which the patient often feels through non-verbal communication [48]. Jarvis et al. found that the mask acted as a stressor in dealing with elderly patients, as they could not see the healthcare worker’s smile, the movement of their lips, the muffled sound, or their facial expressions [49]. Moreover, the tone increase used by the healthcare workers to compensate for these non-verbal cues increases the risk of misinterpretation as showing power, dominance, or abuse, a feeling that only augments the increased powers given to them through the pandemic [49].

Respect in healthcare is focused on honoring the autonomy of patients who have the capacity to make decisions while enhancing the safeguards in place for patients with diminished decision-making abilities [50,51]. Many patients with dementia have decreased and sometimes fluctuating decisional capacity. Respecting their autonomy entails a repeated, multi-step process of evaluating their choices and, whenever decisions are not taken autonomously, involving family members or friends in the decision-making process or even acting without a formal agreement if specific medical procedures are essential for the patient’s well-being. However, as some authors have recently emphasized, respect for patients with dementia has other particular issues, such as the optionality of intentionality as a prerequisite step for validating autonomy or increased ambiguities centering the importance of precedent autonomy over beneficence [52]. The latter is especially important in association with COVID, as autonomy was potentially limited anyway through quarantine and isolation, which were often not very well (or at all) understood by this social group, increasing the contagion risk for others, including family members and friends. Dementia is known to cause behavioral changes such as wandering and agitation, reducing compliance with quarantine or isolation measures, and increasing the risk of disease transmission [53]. This hypothesis was supported by large-scale clinical studies showing that patients with dementia are more likely to contract the disease compared to people without it [54]. It can also impair executive function, potentially affecting understanding and compliance with public health recommendations such as social distancing, mask-wearing, glove-wearing, and regular handwashing [55]. COVID-19 has also been shown to increase depression symptoms and the use of antidepressant, antipsychotic, opioid, and antianxiety drugs [56], further affecting decisional capacity and compliance with isolation/quarantine.

Caring implies showing kindness and being motivated to aid patients with dementia [47]. This axis was perhaps the most affected during the COVID-19 pandemic. Many studies have shown that healthcare workers’ fears have decreased or altered the care given to COVID-19 patients [57,58,59]. This has been especially noticed in the elderly, including dementia patients, as they are often deprioritized from life-saving medical procedures compared to other social groups [33,60,61]. Patients with dementia may have increased anxiety due to social distancing, which was an essential part of the protocols recommended by various national and international health organizations [62], further decreasing their perception of medical care.

Creating a humane and purposeful environment involves directing efforts toward cultivating a welcoming and recognizable atmosphere, which can enhance a sense of safety and independence for patients [63]. During the COVID-19 pandemic, this desideratum was often seen as unrealistic, as the main focus of healthcare services was on optimizing the allocation of highly finite resources toward a maximum number of patients. This approach was shown to cause deconditioning, decrease formal and informal care support, and increase social isolation [64], further increased by the extensive usage of telehealth services, to which this group of patients was unfamiliar and unable to adapt. For example, Gately et al. found the following barriers to using telehealth in patients with significant cognitive declines: lower levels of device ownership, limited access to adequate internet, language barriers, difficulties in managing technical issues, challenges in learning the required technology, and decreased baseline comfort in using these technologies [65]. Other studies have shown, on the contrary, that these services are valuable and well-received by these patients, as they augment their quality of life through increased (even though mediated) social interaction and increased participation in tasks that benefit their health [66,67]. Still, their advantages were only manifested if the patients were assisted in using these technologies correctly.

Exerting a degree of mild restraint to meet the person’s essential needs [18] can apparently be seen as opposed to respecting one’s dignity. Restraining a person’s free movement is a de facto breach of their autonomy. In the case of patients with dementia, the use of restraints may serve a distinctly advantageous purpose for both the individual by reducing the likelihood of self-injury and for society by restricting their mobility and thereby decreasing the potential for them to transmit the virus. To be ethically acceptable, restraints should be minimal in their purpose and only employed if other methods of minimizing harm cannot be utilized effectively. If they are employed, we should also consider some of their side effects, such as increased risk of aspiration pneumonia, deep venous thrombosis, or pulmonary embolism [68], which are also potential complications of COVID-19 and/or might increase the risk for an unfavorable course of the respiratory or cardiovascular dysfunction associated with this disease. During the COVID-19 pandemic, the use of physical restraints was augmented, and not only for the benefit of the patients. For example, Okuno et al. demonstrated that the lowered threshold for employing physical restraints on COVID-19 patients with dementia has been influenced by the heightened physical and psychological strain experienced by healthcare personnel during the pandemic, as well as the adoption of restrictive hospital policies to manage its impact [68]. Other authors have argued that the use of restraints was caused by restrictive hospital visitation hours, leading to increased social isolation and decreased chances for caregiver advocacy [69,70].

Minimizing harm in terminal care entails respecting patients’ preferences regarding death and dying through advance care planning [34], optimizing terminal and palliative care [71,72], and respecting the principle of justice by avoiding discrimination of any kind, especially age-based [33]. According to Weisman, dying well needs proper management of four main issues from the patients: understanding that they are dying, reaching a significant level of acceptance, feeling that death is timely, and compliance with the terms of dying from both a personal and a relational (group) level [73]. The approaches presented in the literature toward minimizing harm vary. For example, Lapid et al. found that harm minimization should focus on the following axes in these patients: advanced care planning, symptoms management (including palliative sedation), voluntary-assisted dying and euthanasia, improving access to long-term care facilities, and management of hospice and in-home dying [34]. Liu et al. argued, in a consensus paper regarding dementia well-being and COVID-19, that passing well should be the last step in the management of these patients, following preventing well, diagnosing well, treating well, supporting well, and living well, and should entail making difficult decisions more quickly and developing decision aids for both the patients and their families [74]. Parks and Howard emphasized the need for analyzing death during COVID-19 through the lens of relational autonomy, needing to consider not only the maximization of prevention through social isolation but also the needs of the families and patients for social interaction [75].

Justice was one of the most analyzed ethical issues concerning COVID-19, from prioritization rules for vaccination [36,76,77] and research [78] to issues appertaining to resource allocation in various vulnerable groups or discrimination [33,79,80]. Patients with dementia were critically vulnerable concerning resource allocation during the pandemic. This vulnerability was caused not only by the disease but also by some of their characteristics, both physiological (such as advanced age, which was used formally or informally as a tool to exclude some patients from certain types of scarce interventions) and pathological (significant associated comorbidities leading to a decreased prioritization). For example, in Switzerland, initial guidelines from 2020 limited access to the ICU from the triage stage to patients with moderate and severe dementia. As this approach was seen as discriminatory, this exclusion was removed later, and they introduced a score entitled the Clinical Frailty Scale, aimed at evaluating short-term prognosis. This was also seen as indirectly discriminatory because the way it was built severely limited access to the ICU, even to patients with moderate dementia, due to their need for aid due to their neurological and psychiatric status [81]. Some ways the principle of justice was applied during the COVID-19 pandemic and the impact on patients with dementia are presented in Table 1 below.

**Table 1:** Allocation approaches and their effects on patients with dementia (based on [20,33,76,82]).

Approach	Advantages	Disadvantages	Effects on Patients with Dementia
Prioritizing patients without comorbidities	Maximization of therapeutic benefits Increased number of QALY and DALY	Increased burden on patients from vulnerable groups, mainly the elderly, poor, or those with multiple comorbidities	Significantly decrease healthcare access (most have multiple additional comorbidities are in the elderly age groups)
Maximizing QALY	Increases the number of years lived with optimal quality	Difficult to implement in triage/I.C.U. environments Prioritizes young and healthy patients It does not take into account the QALY as perceived by the patient	Significantly decrease healthcare access (physicians are sometimes biased in seeing people with dementia as having a lower quality of life; patients are older, with multiple comorbidities)
Prioritizing young patients	Increases the number of years of life saved Younger patients have an increased chance of reaching an advanced age It is allowed if it is non-discriminatory (based on morally relevant criteria, such as objective clinical scores) Sometimes, even older adults want younger patients to be prioritized It is easy to use in emergency settings	Discriminatory against older people based on a morally irrelevant criterion (age) Seen as ageist The apparent acceptance of this approach by older adults is relative, being potentially caused by ageist attitudes within society or even the medical profession It might lead to other controversial strategies (based on the slippery slope argument)	Significantly decrease healthcare access through: deprioritization during triage reduces addressability of patients to healthcare units (the patients/caregivers see it as futile accessing healthcare units if they are not to be treated, preferring to remain to die at home)
First came the first server	Egalitarian	It can deprioritize patients with significantly higher chances of survival It can be seen as wasteful from a resource allocation perspective It can generate difficulties in withdrawing interventions with a relatively minor indication or even futile	Does not cause a decrease in healthcare access
Prioritization of healthcare/critical personnel	Increases the operational response during the pandemic Respects the principle of reciprocity (increased burden and risks lead to increased benefits).	It is hard to establish what personnel are deemed critical It is a non-medical criterion Decreases trust in the medical profession	It may cause a deprioritization of patients with dementia, but this is in line with other non-critical patients. The actual effect depends on the second-tier criteria used for triage.
Prioritization of vaccinated patients	Increases the motivation for vaccination	Decreases the addressability of patients during the early stages of the disease It may be seen as discriminatory (refusal to vaccinate may be seen as a signal for information deficits or lower socio-economic status)	Does not deprioritize patients with dementia directly.

Another issue that has recently been discussed regarding human dignity at a societal level (based on the societal level of the Ring Theory of Personhood) is its application at a collective level, generated by the presence of a series of obligations and responsibilities to each citizen caused by their belonging to a particular nation [83]. The COVID-19 pandemic has generated many challenges that must be addressed on each level, from the innate to the societal. On a societal level, the obligations and responsibilities of dementia patients were sometimes enforced with little consideration for their dignity as human beings, motivated by the need to prevent the spread of the disease. The main issue reported in the literature is a decreased understanding of what COVID-19 is and how it may be prevented, leading to an increased difficulty in risk-appraisal of their actions [84].

## 5. Limits of the Study

This is a qualitative analysis of the concept of dignity as it was evaluated in association with dementia. Quantitative analyses should be performed to better assess how it is implemented and respected in clinical practice. Being an overlooked principle, dignity was often briefly evaluated in other authors’ studies, limiting our possibilities to properly analyze its potential implications in clinical practice. The concept of dignity in itself lacks the standardization of other ethical principles, making ethical analyses even trickier.

## 6. Conclusions

In periods of significant stress and burden, the well-being of patients and their rights had to be balanced in a way that did not fully respect the accepted approach to morality in medical practice. Irrespective of new developments or threats, medicine should always center its attention on the patient and respect his rights and dignity as a human being.

Dignity is a multifaceted concept in dementia patients, the dimensionality being only further augmented by the interposition of COVID-19, which, at its beginnings, had many unknowns that forced the healthcare systems, healthcare workers, patients, and society, in general, to act based on many unfamiliar elements in a way that, from an outside/unbiased perspective, may seem excessive.

Applying this principle in clinical practice requires significant commitment from all healthcare workers. New approaches to the analysis of dignity, such as through the Ring Theory of Personhood, may facilitate its understanding by practitioners and aid its implementation in populations with multiple vulnerabilities, such as dementia patients, during an infectious outbreak that generates significant social and medical changes.

## Data Availability

Not applicable.
